# Subclinical Inflammation and Endothelial Dysfunction in Young Patients with Diabetes: A Study from United Arab Emirates

**DOI:** 10.1371/journal.pone.0159808

**Published:** 2016-07-26

**Authors:** Elhadi H. Aburawi, Juma AlKaabi, Taoufik Zoubeidi, Abdullah Shehab, Nader Lessan, Awad Al Essa, Javed Yasin, Hussain Saadi, Abdul-Kader Souid

**Affiliations:** 1 Department of Pediatrics, College of Medicine and Health Sciences, UAE University, Al Ain, United Arab Emirates; 2 Department of Internal Medicine, College of Medicine and Health Sciences, UAE University, Al Ain, United Arab Emirates; 3 Department of Mathematics, College of Sciences, UAE University, Al Ain, United Arab Emirates; 4 Department of Internal Medicine, Imperial College London Abu Dhabi, Abu Dhabi, United Arab Emirates; 5 Cleveland Clinic, Abu Dhabi, United Arab Emirates; University of Catanzaro Magna Graecia, ITALY

## Abstract

**Background:**

The impact of obesity and dyslipidemia on cardiovascular health in adolescents and young adults with diabetes is incompletely understood. This study evaluated the effects of these co-morbidities on markers of inflammation and endothelial dysfunction in young patients with the disease.

**Methods:**

The study investigated sets of inflammatory, endothelial, and adipocyte biomarkers in 79 patients with type 1 diabetes, 55 patients with type 2 diabetes, and 47 controls.

**Results:**

Mean (±SD) age was 20±6 y (median = 17, range = 12–31). Patients with diabetes had higher levels of cytoadhesive molecules (sICAM-1 and sVCAM-1, *p*<0.001), adiponectin (*p*<0.001), and haptoglobin (*p* = 0.023). Their heart rate variability assessment revealed lower standard deviation of beat-to-beat intervals and lower total power (*p*≤0.019), reflecting autonomous nervous dysfunction. Hemoglobin A1c >8.0% (estimated average blood glucose >10 mmol/L) was associated with higher adiponectin (*p*<0.001) and obesity was associated with lower adiponectin (*p*<0.001); thus, obesity damped the effect of hyperglycemia on adiponectin. Obesity was associated with higher sICAM-1 (*p*≤0.015), tumor necrosis factor-α (TNFα), interleukin-6 (IL-6), and high-sensitivity C-reactive protein (hs-CRP), *p*<0.001. Similarly, high-density lipoprotein (HDL) <1.02 mmol/L was associated with higher sICAM-1, TNFα, IL-6, and hsCRP (*p*≤0.009) and lower adiponectin (*p*<0.001). Adiponectin correlated negatively with the inflammatory biomarkers in patients with diabetes.

**Conclusion:**

Subclinical inflammation and endothelial dysfunction are common among young patients with diabetes. Poor diabetes control is associated with higher adiponectin. Obesity and dyslipidemia are associated with lower adiponectin and higher inflammatory and endothelial biomarkers. Intuitively, these predictors of cardiovascular disease are amenable to proper glycemic control, nutritional choices, and regular exercise.

## Introduction

The rising rates of diabetes, obesity, atherogenic dyslipidemia, and cardiovascular disease (stroke and ischemic heart disease) impose serious health problems worldwide [1–2]. Children and young adults are especially vulnerable to complications of these disorders and are generally less engaged in health promoting and monitoring programs. Therefore, measures designed for preventing and treating obesity, dyslipidemia, and hypertension in children, adolescents, and young adults with diabetes are vital.

This study investigated sets of inflammatory and endothelial dysfunction biomarkers in young patients with diabetes. Its main purpose was to use established predictors of microvascular disease (tumor necrosis factor-α [TNFα], interleukin-6 [IL-6], high-sensitivity C-reactive protein [hs-CRP], soluble intercellular cytoadhesive molecule-1 [sICAM-1], soluble vascular cytoadhesive molecule-1 [sVCAM-1], and adiponectin) as screening tools for adverse effects of obesity and dyslipidemia in this age group [3–4]. ICAM-1 is a glycoprotein involved in tissue adhesion and is expressed in response to cytokines [5] and therefore, it has been used as a biomarker for inflammation [6]. Its circulating soluble form (sICAM-1) in the blood (normal, 150 ± 32 ng/mL) estimates levels in the tissue [5–6]. Adiponectin reduces free fatty acid levels and promotes lipid metabolism. This cardio-protective, adipocyte-derived cytokine improves insulin function and ameliorates inflammation and atherogenic disease [7]. Adiponectin is also known to modulate endothelial function [8]. Despite its proven importance, adiponectin is yet to be included in routine patient care.

## Methods

This study involved UAE citizens (12 to 31 years of age) with diabetes. The study was approved by Al Ain Medical District Human Research Ethics Committee (AAMDHR 09/79) and Imperial College London Diabetes Centre Research Ethics Committee (REC 017). Written informed consent was obtained for each participant (or their parents if they were <18 years old) prior to study enrolment.

Patients were randomly recruited from three diabetes centers (Tawam Hospital, Al Ain Hospital, and Imperial College London Diabetes Centre) in Abu Dhabi region. The study had a two-stage sampling design, stratified by center and systematic sampling from each center. Even-numbered patients on appointment lists were selected. Healthy citizens (12 to 18 years of age) were also recruited from public schools and healthy citizens between 18 and 31 years were recruited from the UAE University. Following written approval from the UAE University, students were invited by email to take part in this study and those who responded positively were randomly selected from several colleges. School students were also selected randomly, using multistage sampling. Firstly, four male and four female schools were selected randomly from a list of middle and secondary schools from the list provided by Abu Dhabi Education Council. Secondly, 2 classes from each grade were selected randomly from these schools. Thirdly, all students from these classes were approached to participate in the study and those who signed the study informed consent were enrolled. Assessments were performed at the diabetes centers (patients), schools (controls), and university (controls).

Patients with type 1 (T1DM) or type 2 (T2DM) diabetes mellitus were included. Exclusion criteria were active infection, chronic illness (e.g., rheumatoid arthritis, hyperthyroidism, and inflammatory bowel disease), regular use of certain medications (β-blockers, α-blockers, diuretics, and hormone therapies), pregnancy, and inability to give informed consent.

Patients with T1DM were receiving daily insulin since diagnosis; two patients were also receiving atorvastatin and one patient was receiving ezetimibe. Medications used by patients with T2DM included metformin (22 patients), metformin plus sitagliptin (three patients), metformin plus vildagliptin (one patient), sulfonylurea (nine patients), diet plus statin (nine patients), insulin (five patients), and diet plus exercise (six patients). Control participants were not receiving any regular medication.

All participants completed the study health questionnaire, physical examination, and laboratory assessments. They were interviewed by a trained nurse who performed examination and anthropometric measurements (weight, height, waist, and hip circumference). Fat mass and fat-free mass were measured using Tanita body composition analyzer (Tanita Corporation, Tokyo, Japan). Abnormal percent body fat (‘obese’) was set as ≥32% for females and ≥25% for males; otherwise, the values were considered ‘average’ [9]. Heart rate variability (HRV) was performed as previously reported [10]. HRV was recorded for 5 min using a handheld HRV device (Daily Care BioMedical Inc., Taiwan). Measurements were taken over 5 min after 20 min rest, with patient in supine position. Recordings were transferred to computer and data were automatically analyzed by HRV analysis software. Length of recording was selected in accordance with recommendations from the Task Force of the European Society of Cardiology and the North American Society of Pacing Electrophysiology (10). The ratio of power of low frequency band (ms^2^) to power of high frequency band (ms^2^), a measure of the overall balance between sympathetic and parasympathetic systems, was reported. Higher values reflected domination of the sympathetic system and lower ones reflected domination of the parasympathetic system. Total power (reflecting the overall autonomic activity) and SDNN (standard deviation of beat-to-beat, NN, intervals) were determined as previously described [10].

Weight was measured to the nearest 0.1 kg and height to the nearest 0.1 cm in a standing position without shoes and in light clothing using digital scales. Waist circumference was measured with upstretched tapes midpoint between the bottom of the rib cage and the tip of the iliac crest. Hip circumference was measured over minimal clothing at the level of the greatest protrusion of the gluteal muscles. Blood pressure was measured on the right arm at rest for ≥5 min. Three consecutive measures were obtained at one-minute intervals with a standard mercury sphygmomanometer with an appropriate cuff size.

Blood samples were collected after an overnight fast and processed for glucose, HbA1c, low-density lipoprotein (LDL), high-density lipoprotein (HDL), triglycerides, total cholesterol, interleukin-6 (IL-6), tumor necrosis factor-α (TNFα), high-sensitivity C reactive protein (hsCRP), soluble intercellular cytoadhesive molecule-1 (sICAM-1), soluble vascular cytoadhesive molecule-1 (sVCAM-1), and adiponectin. Urine samples were analyzed for creatinine, isoprostane and protein. Protein:creatinine ratio <15 mg/mmol was considered normal.

Glucose, hemoglobin A1c (HbA1c), lipid profile, and creatinine were measured using an automated analyzer Integra 400 Plus (Roche Diagnostics, Mannheim, Germany). 25-Hydroxy-vitamin D was measured by a chemi-luminescent assay with the automated analyzer Cobas e411 (Roche Diagnostic, Mannheim, Germany). The laboratory performed internal quality controls before running samples and participated in External Quality Assurance program through the College of American Pathologists Proficiency Testing.

Enzyme linked immunosorbent assays from R&D Systems were used to measure adiponectin (Acrp30 Quantikine, DRP300), IL-6 (Human IL-6 Quantikine HS, HS600B), TNF*α* (Human TNF*α* Quantikine, DTA00C), sICAM-1, and sVCAM-1 following the manufacturers’ protocols. hsCRP was measured using Synchron Clinical System (UniCel DxC-800) from Beckman Coulter, Inc. (Fullerton, CA, USA).

The statistical analysis was performed using SPSS software version 21.0 (SPSS Inc., Chicago, USA). Measurements were compared across two groups using the Mann-Whitney-U test whenever the group sizes were small (<30) and the values were not normally distributed; otherwise, the independent sample t-test was used. Multiple groups (≥3) were compared using Kruskal-Wallis test whenever measurements were either not normally distributed or heteroscedastic (unequal variances); otherwise, one-way ANOVA was used. Independent-samples t-test (2-tailed, equal variances not assumed), one-way ANOVA (between groups), and nonparametric test (2 independent variables) Mann-Whitney-U test were used to compare groups. The normality and heteroscedasticity of the measurements were tested using Shapiro-Wilk test and Levene test, respectively. Data are presented as mean ± standard deviation (median) with *P*<0.05 (2-tailed) considered significant.

## Results

One hundred and eighty-one subjects were recruited. Sixty four percent (116/181) of the participants had positive family history of diabetes, 42% (77/181) had positive family history of cardiovascular disease, and 6% (11/181) reported high blood pressure (two with T1DM, five with T2DM, and four controls). The anthropometric, laboratory, and clinical measurements are shown in Table 1. In patients with diabetes, the heart rate variability test revealed lower standard deviation of beat-to-beat intervals (SDNN) and lower total power, reflecting autonomic nervous dysfunction (Table 1). Although not significantly different between groups (*p* = 0.117), the LF:HF ratio between power of low and high frequency bands was higher in patients with diabetes compared to those in controls, indicating sympathetic domination (Table 1). SDNN correlated with sVCAM-1 (correlation coefficient, *r* = -230, *p* = 0.028). Patients with diabetes also had higher sICAM-1, sVCAM-1, haptoglobin, adiponectin, triglycerides, total cholesterol, LDL, ALT, and 25-hydroxyvitamin D compared to non-diabetic controls (Table 1). Therefore, aberrant cardiac markers and biochemical disturbances were present in these young patients with diabetes.

**Table 1:** Anthropometric and other measurements in all participants.

	*Non-diabetic controls (n = 47)*	*Type-1 diabetes (n = 79)*	*Type-2 diabetes (n = 55)*	P*
*Anthropometric measurements*				
Age (y)	17.5 ± 4.6 (17.0)	18.6 ± 4.8 (17.0)	23.3 ± 5.8 (24.0)	<0.001
Female: male ratio	22/25	35/44	28/29	0.855^a^
Duration of diabetes (y)	-	6.8 ± 4.1 (6.0)	4.3 ± 3.1 (3.0)	<0.001
Waist circumference (cm)	90.7 ± 19.7 (86.5)	83.3 ± 14.2 (82.0)	101.6 ± 14.3 (103.0)	<0.001
Hip circumference (cm)	103.4 ± 14.5 (104.0)	96.4 ± 14.5 (94.0)	109.8 ± 19.6 (111.0)	<0.001
BMI as adults (kg/m^2^)	25.2 ± 7.6 (23.9)	24.3 ± 6.9 (22.5)	32.0 ± 7.2 (31.5)	<0.001
Body fat (%)	23.2 ± 13.6 (22.7)	20.7 ± 10.4 (21.4)	32.4 ± 10.3 (33.5)	<0.001
Systolic BP (mm Hg)	123 ± 15 (124)	117 ± 13 (116)	117 ± 12 (118)	0.049
Diastolic BP (mm Hg)	74 ± 10 (76)	73 ± 11 (72)	74 ± 8 (73)	0.646
*Inflammatory biomarkers*				
sICAM-1 (ng/mL)	184 ± 40 (186)	243 ± 71 (245)	281 ± 102 (257)	<0.001
sVCAM-1 (ng/mL)	570 ± 150 (531)	782 ± 208 (752)	730 ± 220 (745)	<0.001
IL-6 (pg/mL)	1.7 ± 2.1 (1.0)	1.5 ± 1.5 (0.9)	2.5 ± 2.4 (1.8)	0.015
Haptoglobin (mg/dL)	116 ± 54 (105)	139 ± 60 (139)	150 ± 68 (145)	0.031
hsCRP (mg/L)	3.4 ± 5.1 (1.6)	3.9 ± 4.3 (2.5)	5.1 ± 6.0 (3.2)	0.148
TNF*α* (pg/mL)	1.9 ± 2.0 (1.1)	1.8 ± 1.5 (1.2)	2.1 ± 1.7 (1.4)	0.437
Isoprostane (pg/mL)	2.4 ± 1.7 (2.2)	3.4 ± 2.6 (3.0)	3.7 ± 3.0 (2.8)	0.075
*Lipid profiles*				
Adiponectin (μg/L)	3.4 ± 1.2 (3.2)	10.5 ± 7.1 (9.8)	5.2± 3.2 (4.1)	<0.001
HDL (mmol/L)	0.9 ± 0.2 (0.9)	1.3 ± 0.4 (1.2)	1.0 ± 0.3 (0.9)	<0.001
Triglycerides (mmol/L)	1.0 ± 0.7 (0.8)	1.4 ± 0.8 (1.1)	1.7 ± 1.2 (1.4)	<0.001
Total cholesterol (mmol/L)	4.0 ± 0.8 (3.9)	4.7 ± 1.1 (4.6)	4.3 ± 1.1 (4.2)	0.003^b^
LDL (mmol/L)	2.4 ± 0.7 (2.4)	2.8 ± 0.9 (2.7)	2.5 ± 1.0 (2.5)	0.048
*Metabolic*, *hepatic*, *and renal markers*				
Hemoglobin A1c (%)	-	9.4 ± 2.1 (9.5)	7.8 ± 2.5 (7.4)	<0.001
Blood glucose (mmol/L)	-	15.3 ± 8.4 (13.6)	9.5 ± 6.3 (7.6)	<0.001
25-Hydroxyvitamin D (ng/mL)	12.6 ± 7.7 (10.5)	18.8 ± 9.0 (17.4)	18.7 ± 10.8 (15.7)	<0.001
ALT (U/L)	-	20.8 ± 14.3 (16.6)	37.7 ± 31.2 (27.4)	0.001
GGT (U/L)	-	20.6 ± 22.7 (13.5)	27.6 ± 13.9 (27.0)	0.123
Protein:creatinine (mg/mmol)	1.7 ± 2.2 (0.6)	1.5 ± 1.3 (1.1)	1.1 ± 0.9 (0.8)	0.072
*Cardiac markers*				
SDNN (ms)	73 ± 45 (63)	51 ± 29 (44)	55 ± 47 (43)	0.011
Total power (ms^2^)	6021±16726 (1816)	1630±2163 (878)	1651±2954 (991)	0.019
LF:HF ratio	1.7 ± 0.9 (1.4)	2.7 ± 2.0 (2.0)	2.5 ± 2.0 (1.9)	0.117
QTc (ms)	427 ± 71 (427)	427 ± 73 (412)	421 ± 63 (414)	0.689

Values are mean SD (median). Isoprostane was measured in the urine; all other measurements were in the serum. A few measurements were missing in some patients.

* Kruskal Wallis test (between groups).

^a^ Pearson’s chi-square test.

^b^ One-way ANOVA.

BP, blood pressure; IL-6, interleukin-6 (normal median level, 1.45 pg/mL); TNF*α*, tumor necrosis factor-alpha (pg/mL); hsCRP, high sensitivity C-reactive protein (low, <1.0 mg/L; average, 1–3 mg/L; high, >3.0 mg/L); sICAM, soluble intercellular cell adhesion molecule-1 (normal, 265 ± 55 ng/mL [20]); sVCAM-1, soluble vascular cell adhesion molecule-1 (normal, 408 ± 76 ng/mL [2]); HDL, high-density lipoprotein; LDL, low-density lipoprotein; GGT, gamma-glutamyl transferase (normal, 9–58 U/L), ALT, alanine aminotransferase (normal, 7–21 U/L); QTc, corrected QT interval (a measure of myocardial repolarization); SDNN, standard deviation of beat-to-beat (NN) intervals (intervals <35 ms correlate with autonomous nervous system dysfunction; normal mean value at 10–20 y = 55 ms and 20–30 y = 47 ms); LF/HF, ratio between the power of low frequency (ms^2^) and high frequency (ms^2^) bands (reference values, 0.5–2.0; it is a measure of the overall balance between sympathetic and parasympathetic systems). Higher LF:HF ratios reflect domination of the sympathetic system and lower ones reflect domination of the parasympathetic system; total power reflects the overall autonomic (mainly sympathetic) activity (values ≤1,000 ms^2^ suggest autonomous nervous system dysfunction).

Protein: creatinine ratio <15 mg/mmol are considered normal. Percent body fat ≥25 in men and ≥32 in women is considered obese. Overweight is defined as BMI ≥25 kg/m^2^ and obesity ≥30 kg/m^2^. Normal haptoglobin levels are 41–165 mg/dL. At any age, HDL is considered normal if >1.17 mmol/L and low if <0.91 mmol/L. Normal LDL levels are <2.85 mmol/L. Total cholesterol ≥5.18 mmol/L is definitely high and needs treatment. Triglycerides levels ≥1.5 mmol/L in patients 10–19 y are considered high.

Patients with HbA1c >8% (estimated average blood glucose of >10 mmol/L) had higher adiponectin levels than those with HbA1c ≤8% (*p*<0.001); otherwise, the biomarkers of endothelial dysfunction and inflammation were not significantly different between the two subgroups (Table 2). Obesity (BMI >30 kg/m^2^ or body fat ≥32% in females or ≥25% in males) was associated with higher sICAM-1 (*p*≤0.015), TNFα, IL-6, and hsCRP (*p*<0.001). Obesity was also associated with lower adiponectin (*p*≤0.002). Similarly, HDL <1.02 mmol/L was associated with higher sICAM-1 (*p* = 0.009), TNFα (*p* = 0.005), IL-6 (*p* = 0.004), and hsCRP (*p* = 0.006); and lower adiponectin (*p*<0.001). LDL >2.9 mmol/L was associated with higher TNF*α* (*p* = 0.005) and hsCRP (*p* = 0.007). Triglycerides >1.2 mmol/L and total cholesterol >4.5 mmol/L were associated with higher inflammatory biomarkers (*p*≤0.021), Table 2.

**Table 2:** Effects of hyperglycemia and excess body fat on the endothelial dysfunction and inflammatory biomarkers and adiponectin in patients with T1DM or T2DM.

	*Cutoffs*	n*		*sICAM -1(ng/mL)*	*sVCAM-1(ng/mL)*	*TNFα (pg/mL)*	*IL-6 (pg/mL)*	*hsCRPmg/L*	*Adiponectin (μg/L)*
HbA1c (%)	>8%	49		247 ± 84 (250)	753 ± 234 (745)	1.8 ± 1.5 (1.2)	1.7 ± 1.5 (1.2)	4.1 ± 4.2 (3.0)	10.3 ± 7.3(9.4)
	≤8%	39		274 ± 91 (258)	766 ± 229 (791)	1.7 ± 1.4 (0.9)	2.2 ± 2.7 (1.0)	3.7 ± 4.8 (1.4)	6.0 ± 3.5 (4.6)
			*P*	-	-	-	-	-	0.001
BMI (kg/m^2^)	>30	47		294 ± 103 (270)	727 ± 217 (713)	2.7 ± 1.6 (2.2)	3.1 ± 2.3 (2.7)	6.7 ± 5.6 (5.4)	5.5 ± 3.6 (4.1)
	≤30	75		239 ± 70 (237)	778 ± 211 (762)	1.5 ± 1.4 (1.0)	1.2 ± 1.4 (0.8)	3.0 ± 4.3 (1.3)	9.8 ± 6.9 (7.9)
			*P*	0.002	-	<0.001	<0.001	<0.001	<0.001
Body fat (%)	Obese^¶^	32		274 ± 79 (260)	720 ± 147 (705)	3.1 ± 1.9 (3.0)	2.4 ± 1.6 (2.0)	7.5 ± 6.2 (5.5)	6.4 ± 4.7 (4.8)
	Average	47		234 ± 64 (229)	802 ± 219 (762)	1.8 ± 0.9 (0.9)	0.9 ± 0.9 (0.5)	1.9 ± 2.1(1.2)	10.5 ± 6.9 (10.4)
			*P*	0.015	0.069	<0.001	<0.001	<0.001	0.002
HDL (mmol/L)	<1.02	52		280 ± 109 (262)	732 ± 211 (712)	2.4 ± 1.8 (1.9)	2.2 ± 1.7 (1.9)	5.7 ± 6.3 (4.0)	5.3 ± 3.6 (4.0)
	≥1.02	45		233.4± 58 (233)	772 ± 193(749)	1.5 ± 1.2 (0.9)	1.3 ± 1.5 (0.7)	2.9 ± 3.1 (1.3)	11.0 ± 6.3 (10.4)
			*P*	0.009	-	0.005	0.004	0.006	<0.001
LDL (mmol/L)	>2.9	45		276 ± 98 (268)	796 ± 201 (791)	2.5 ± 1.9 (1.9)	2.3 ± 2.0 (1.9)	6.2 ± 5.9 (4.6)	9.3 ± 7.6 (5.6)
	≤2.9	77		251 ± 81 (237)	737 ± 219 (745)	1.6 ± 1.3 (1.1)	1.8 ± 2.0 (1.0)	3.4 ± 4.4 (1.4)	7.5 ± 5.2 (5.7)
			*P*	-	-	0.005	-	0.007	-
Triglycerides(mmol/L)	>1.2	59		275 ± 108 (260)	732 ± 206 (712)	2.3 ± 1.7 (1.9)	2.4 ± 2.0 (1.9)	5.8 ± 5.9 (3.9)	7.2 ± 6.9 (4.6)
	≤1.2	63		246 ± 61 (237)	783 ± 220 (766)	1.6 ± 1.4 (1.0)	1.5 ± 1.9 (0.8)	3.2 ± 4.0 (1.3)	9.0 ± 5.5 (8.0)
			*P*	0.074	-	0.016	0.012	0.006	-
Total cholesterol (mmol/L)	>4.5	57		269 ± 94 (260)	778 ± 208 (764)	2.2 ± 1.6 (1.8)	2.2 ± 2.0 (1.8)	5.4 ± 5.2 (3.9)	9.3 ± 7.5 (7.6)
	≤4.5	63		253 ± 84 (245)	749 ± 218 (748)	1.6 ± 1.3 (1.0)	1.7 ± 2.0 (0.9)	3.3 ± 4.2 (1.3)	7.2 ± 4.7 (5.2)
			*P*	-	-	0.015	-	0.021	-

*P*-values are independent-samples T test (2-tailed); only significant or near significant levels are shown.

* Measurements were missing for some patients.

^¶^ Obesity was defined as body fat ≥32% for females or ≥25% for males; otherwise, the values were considered average.

Changes in the inflammatory biomarkers and adiponectin in non-diabetic controls are shown in Table 3. Obesity was associated with higher levels of TNF*α* (*p* = 0.008), IL-6 (*p*<0.001), hsCRP (*p*<0.001), and adiponectin (*p* = 0.005). LDL >2.9 mmol/L was associated with higher IL-6 (*p* = 0.023) and hsCRP (*p* = 0.041). Total cholesterol >4.5 mmol/L was associated with higher TNF*α* (*p* = 0.023) and IL-6 (*p* = 0.030), Table 3.

**Table 3:** Effects of excess fat on endothelial and inflammatory biomarkers and adiponectin in control subjects.

	*Cutoffs*	n*		*sICAM-1(ng/mL)*	*sVCAM-1 (ng/mL)*	*TNFα (pg/mL)*	*IL-6 (pg/mL)*	*hsCRPmg/L*	*Adiponectin(μg/L)*
Body fat (%)	Obese^¶^	16		188 ± 32 (190)	560 ± 104 (539)	3.0 ± 2.9 (1.7)	3.0 ± 2.8 (2.0)	6.7 ± 6.8 (4.2)	4.2 ± 1.3 (3.6)
	Average	27		182 ± 45 (178)	576 ± 174 (530)	1.2 ± 0.8 (0.9)	1.0 ± 1.0 (0.9)	1.5 ± 2.0 (0.8)	3.0 ± 0.9 (2.6)
			*P*	-	-	0.008	<0.001	<0.001	0.005
LDL (mmol/L)	>2.9	11		189 ± 38 (187)	511 ± 72 (488)	3.7 ± 3.2 (2.6)	3.4 ± 3.4 (1.9)	7.3 ± 8.3 (3.2)	3.9 ± 1.7 (3.2)
	≤2.9	31		184 ± 41 (186)	596 ± 164 (559)	1.2 ± 0.7 (1.0)	1.2 ± 1.0 (1.0)	2.1 ± 2.3 (1.2)	3.3 ± 1.0 (3.3)
			*P*	-	-	0.052	0.023	0.041	-
Total cholesterol (mmol/L)	>4.5	10		192 ± 42 (179)	507 ± 66 (491)	3.2 ± 2.6 (2.3)	2.9 ± 2.8 (1.6)	5.6 ± 6.3 (2.8)	4.1 ± 1.7 (4.1)
	≤4.5	32		183 ± 39 (189)	595 ± 163 (554)	1.5 ± 1.7 (1.0)	1.4 ± 1.8 (1.0)	2.8 ± 4.6 (1.3)	3.3 ± 1.0 (3.3)
			*P*	-	-	0.029	0.030	0.072	-

*P*-values are from the Mann-Whitney test, except for sICAM-1 which are from the independent-samples T test (2-tailed); only significant or near significant levels are shown.

* Measurements were missing for some patients.

^¶^ Obesity was defined as body fat ≥32% for females or ≥25% for males; otherwise, the values were considered average. At any age, HDL is considered normal if >1.17 mmol/L and low if <0.91 mmol/L. Normal LDL levels are <2.85 mmol/L. Total cholesterol ≥5.18 mmol/L is definitely high and needs treatment. Triglycerides levels ≥1.5 mmol/L in patients 10–19 y are considered high.

sICAM-1, soluble intracellular cell adhesion molecule-1; sVCAM-1, soluble vascular cell adhesion molecule-1; TNF*α*, tumor necrosis factor-alpha; IL-6, interleukin-6; hsCRP, high sensitivity C-reactive protein; LDL, low-density lipoprotein.

Correlations between inflammatory biomarkers and adiponectin are shown in Table 4. In patients with T1DM or T2DM, adiponectin correlated negatively with sICAM-1, TNF*α*, IL-6, and hsCRP (*p*≤0.037). In addition, sICAM-1 correlated positively with TNF*α* (*p* = 0.001), IL-6 (*p* = 0.001), hsCRP (*p* = 0.004), and sVCAM-1 (*p* = 0.010), Table 4. In non-diabetic controls, adiponectin correlated positively with TNF*α*, IL-6, hsCRP, and haptoglobin (*p*≤0.021); and sICAM-1 correlated only with TNF*α* (*p* = 0.004), Table 4.

**Table 4:** Spearman's rho correlations in patients with diabetes and non-diabetic controls.

Patients with T1DM or T2DM (n = 121)							
	sICAM-1	sVCAM-1	TNF*α*	IL-6	HsCRP	Haptoglobin	Adiponectin
sICAM-1	-	0.230 (0.011)	0.258 (0.004)	0.297 (0.001)	0.298 (0.001)	-	-0.199 (0.028)
sVCAM-1	0.230 (0.011)	-	-	-	-	-	-
TNFα	0.258 (0.004)	-	-	0.737 (<0.001)	0.887 (<0.001)	0.737 (<0.001)	-0.189 (0.038)
IL-6	0.297 (0.001)	-	0.737 (<0.001)	-	0.708 (<0.001)	0.535 (<0.001)	-0.227 (0.012)
hsCRP	0.298 (0.001)	-	0.887 (0.000)	0.708 (0.000)	-	0.736 (0.000)	-0.189 (0.037)
Haptoglobin	-	-	0.737 (0.000)	0.535 (0.000)	0.736 (0.000)	-	-
Adiponectin	-0.199 (0.028)	-	-0.189 (0.038)	-0.227 (<0.012)	-0.189 (<0.037)	-	-
Non-diabetic controls (n = 43)							
	sICAM-1	sVCAM-1	TNF*α*	IL-6	HsCRP	Haptoglobin	Adiponectin
sICAM-1	-	0.431 (0.004)	-	-	-	-	-
sVCAM-1	0.431 (0.004)	-	-	-	-	-	-
TNFα	-	-	-	0.963 (<0.001)	0.928 (<0.001)	0.717 (<0.001)	0.457 (0.002)
IL-6	-	-	0.963 (<0.001)	-	0.975 (<0.001)	0.762 (<0.001)	0.390 (0.010)
hsCRP	-	-	0.928 (<0.001)	0.975 (<0.001)	-	0.815 (<0.001)	0.364 (0.017)
Haptoglobin	-	-	0.717 (<0.001)	0.762 (<0.001)	0.815 (<0.001)	-	0.351 (0.021)
Adiponectin	-	-	0.457 (0.002)	0.390 (0.001)	0.364 (0.017)	0.351 (0.021)	-

Numbers are Spearman's rho correlation coefficients. Numbers in parentheses are significance (2-tailed). Only significant correlations are shown. sICAM-1, soluble intracellular cell adhesion molecule-1; sVCAM-1, soluble vascular cell adhesion molecule-1; TNF*α*, tumor necrosis factor-alpha; IL-6, interleukin-6; hsCRP, high sensitivity C-reactive protein.

Distribution of the inflammatory biomarkers, cytoadhesive molecules, and adiponectin among patients and controls and their correlations with each other are shown in S1–S3 Figs. A schematic summary of the results is also shown in S1 File. In all studied participants (patients and controls), TNFα, IL-6, and haptoglobin correlated with hsCRP (*R*^2^≥0.565), S2 Fig. The correlations between isoprostane and sICAM-1 or sVCAM-1 were nonlinear and complex (S2 Fig). In patients with T1D, adiponectin decreased with age (*R*^2^>0.088) and increased with HbA1c (*R*^2^≥0.766), S3 Fig. In all participants, adiponectin increased with sVCAM-1 (S3 Fig).

## Discussion

Diabetes and obesity are common in UAE. Importantly, the prevalence of these disorders is increasing, particularly among children and young adults. To our knowledge, this is the first study that examined inflammation and endothelial dysfunction in this young population.

This study shows that subclinical inflammation and endothelial dysfunction are highly prevalent in adolescents and young adults with diabetes (Table 1). It also shows significant correlations between these markers and excess body fat and dyslipidemia (primarily low HDL), Table 2. In addition, the heart rate variability test demonstrates autonomic nervous system dysfunction (Table 2). Thus, obesity and dyslipidemia impose deleterious effects on the cardiovascular health of young patients with diabetes. These complications are amenable to prevention and management. These findings have important implications in the management of patients with obesity and diabetes and emphasize the need for early interventions. Recommendations should include regular monitoring of body fat accumulation and appropriate lifestyle changes. Prospective follow-up studies, however, are necessary to investigate the usefulness of these biochemical markers in the clinical care of these patients.

In keeping with studies in older patients, this study has shown that adiponectin correlates negatively with inflammatory biomarkers in young patients with diabetes (Table 4). Adiponectin is significantly lower in patients with BMI >30 kg/m^2^, abnormal body fat (obesity), HDL <1.02 mmol/L, and triglycerides >1.2 mmol/L (Table 2). In contrast, this adipocyte-derived cytokine is significantly higher in patients with HbA1c >8% (Table 2). The studied inflammatory and endothelial dysfunction biomarkers, on the other hand, did not significantly differ between patients with HbA1c >8% *vs*. ≤8% (Table 2). Excess body fat (BMI >30 kg/m^2^ or increased percent body fat) is associated with higher sICAM-1, TNF*α*, IL-6, and hsCRP. A similar profile of increased sICAM and inflammatory biomarkers and decreased adiponectin is observed in patients with dyslipidemia (HDL <1.02 mmol/L, LDL >2.9 mmol/L, or triglyceride >1.2 mmol/L (Table 2).

In one study, sICAM-1 and sVCAM-1 were measured in children (mean age, 15 y) with obesity, hypertension, or T1D [4]. Both adhesive molecules were significantly higher in children with obesity, hypertension, or diabetes compared to healthy children (sICAM-1: 314 ± 61 ng/mL *vs*. 265 ± 55 ng/mL; sVCAM-1: 514 ± 187 *vs*. 408 ± 76 ng/mL). sICAM-1 was dependent on BMI and sVCAM-1 was dependent on total cholesterol. The authors concluded “endothelial activation appears in these children” [4]. In another study, sVCAM-1 was higher in patients with diabetes compared to controls (924 ± 148 *vs*. 632 ± 139 ng/mL), and the authors related its increased expression to microvascular complications of diabetes [11]. In the same study, adiponectin was lower in patients with T2DM compared to non-diabetic individuals (2.5 ± 1.4 μg/mL *vs*. 4.5 ± 1.0 μg/mL) [11].

In one study, this adipocyte-derived secretory protein inhibited TNF*α*-induced expression of ICAM-1 and VCAM-1, and its circulating levels were lower in patients with coronary disease [8]. The reduced adiponectin in obesity, thus, promotes inflammatory cytokine-induced expression of cytoadhesive molecules. These results are consistent with our findings that excess body fat and dyslipidemia (low HDL) are associated with decreased adiponectin and increased sICAM and sVCAM (Tables 2 and 4).

Diabetes promotes endothelial cell inflammation. In a meta-analysis study, adiponectin levels were found to be higher in patients with T2DM and microvascular complications, such as albuminuria, neuropathy, and retinopathy [12]. Obesity also imposes this cluster of subclinical inflammation and endothelial dysfunction. Therefore, obesity and dyslipidemia are serious co-morbid conditions in patients with diabetes at any age. Several biomarkers have been developed to monitor these processes, especially in high-risk patients. The current study supports their use in children and adolescents with diabetes.

According to the National Health and Nutrition Examination Survey (1999–2008), over 30% of children are at increased risk of being overweight because of increased sedentary activities and consumption of sweetened drinks [13]. Other common co-morbid conditions include hyperlipidemia, BMI >97^th^ percentile, diabetes, hypertension, and smoking [13]. Guidelines from the National Heart Lung and Blood Institute and the American Academy of Pediatrics recommend screening for hyperlipidemia at 9–11 y and at 17–21 y [14–16]. Acceptable total cholesterol is <4.4 mmol/L, LDL <3.11 mmol/L (preferably <2.85 mmol/L), triglycerides <1.0 mmol/L (for 10–19 y), and HDL >1.17 mmol/L (at any age) [15]. Treatment is recommended for HDL <0.91 mmol/L (at any age), triglycerides >1.5 mmol/L (at 10–19 y), and LDL >3.37 mmol/L (at any age) [16–17]. In the current study, low HDL is associated with increased inflammation, endothelial dysfunction, and low adiponectin (Table 2). In the study of Acute Coronary Syndrome Patients in Middle East (6,266 patients), obesity was the main predictor of low HDL [18]. Moderate weight loss, regular exercise, and cessation of smoking significantly increase HDL [18].

Inflammation is an independent predictor of adverse events associated with diabetes [19–20]. Thus, the observed subclinical inflammation in young patients with diabetes and obesity (Tables 1 and 2) should be considered a sign of the disease and to activate early interventions. In a prospective 6-year follow-up study, biomarkers of inflammation and endothelial dysfunction were independent predictors of cardiovascular events in patients with T2DM with microalbuminuria [19]. TNF*α*, sICAM-1, and sVCAM-1 were either a determinant of or associated with cardiovascular disease all-cause mortality [19]. Children and adolescents with T1D also have significantly higher concentrations of sICAM-1, sVCAM-1, TNF*α*, IL-6 [21].

Metabolic syndrome is considered in patients with at least three of the five International Diabetes Federation criteria: (1) increased waist circumference (central obesity); (2) triglycerides ≥1.7 mmol/L; (3) HDL <1.3 mmol/L; (4) systolic blood pressure ≥130 mmHg or diastolic blood pressure ≥85 mmHg; and (5) fasting blood glucose ≥5.6 mmol/L [22–23]. The current study shows diagnostic criteria for metabolic syndrome are met in about 23% (18/79) of patients with T1DM, 55% (30/55) of patients with T2DM, and 19% (9/47) of the controls.

In conclusion, significant biomarkers of inflammation (TNF*α*, IL-6, hsCRP, and haptoglobin) and endothelial dysfunction (sICAM-1 and sVCAM-1) are present in young patients with diabetes, obesity, and dyslipidemia. sICAM-1 is increased in patients with diabetes and correlates with other inflammatory biomarkers. The development of obesity and dyslipidemia needs to be carefully monitored and promptly treated in children and adolescents with diabetes.

## Supporting Information

S1 Fig Endothelial and inflammatory biomarkers in the controls and patients with type-1 or type-2 diabetes. The nonparametric test (2 independent variables; Mann-Whitney) was used to compare between the controls and patients with type-1 (T1DM) or type-2 (T2DM) diabetes mellitus. Isoprostane was measured in the urine; all other measurements were in the serum. Horizontal lines are mean.
(DOCX)

S2 Fig Correlations between studied inflammatory biomarkers in all participants (n = 181). The lines are best fits.
(DOCX)

S3 Fig Adiponectin levels as functions of age, HbA1c, and sVCAM-1 in the controls and patients with type-1 (T1DM) and type-2 (T2DM) diabetes mellitus. The lines are best fits.
(DOCX)

S1 File Schematic presentation of inflammatory and endothelial dysfunctions in young patients with diabetes. (DOCX)
