# Supplementary material for: Subclinical Inflammation and Endothelial Dysfunction in Young Patients with Diabetes: A Study from United Arab Emirates
Source: PLoS One. 2016 Jul 26;11(7):e0159808. doi: 10.1371/journal.pone.0159808 (PMC4961363; doi:10.1371/journal.pone.0159808)
Supplement: S1 Fig — The nonparametric test (2 independent variables; Mann-Whitney) was used to compare between the controls and patients with type-1 (T1DM) or type-2 (T2DM) diabetes mellitus. Isoprostane was measured in the urine; all other measurements were in the serum. Horizontal lines are mean. (DOCX) [file pone.0159808.s001.docx]

|  |  |  |
| --- | --- | --- |
|  |  |  |

**S1 Fig. Endothelial and inflammatory biomarkers in the controls and patients with type-1 or type-2 diabetes.** The nonparametric test (2 independent variables; Mann-Whitney) was used to compare between the controls and patients with type-1 (T1DM) or type-2 (T2DM) diabetes mellitus. Isoprostane was measured in the urine; all other measurements were in the serum. Horizontal lines are mean.
