# Supplementary material for: Subclinical Inflammation and Endothelial Dysfunction in Young Patients with Diabetes: A Study from United Arab Emirates
Source: PLoS One. 2016 Jul 26;11(7):e0159808. doi: 10.1371/journal.pone.0159808 (PMC4961363; doi:10.1371/journal.pone.0159808)
Supplement: S2 Fig — The lines are best fits. (DOCX) [file pone.0159808.s002.docx]

|  |  |
| --- | --- |
|  |  |

**S2 Fig. Correlations between studied inflammatory biomarkers in all participants (n=181).** The lines are best fits.
