# Supplementary material for: Subclinical Inflammation and Endothelial Dysfunction in Young Patients with Diabetes: A Study from United Arab Emirates
Source: PLoS One. 2016 Jul 26;11(7):e0159808. doi: 10.1371/journal.pone.0159808 (PMC4961363; doi:10.1371/journal.pone.0159808)
Supplement: S3 Fig — The lines are best fits. (DOCX) [file pone.0159808.s003.docx]

|  |  |  |
| --- | --- | --- |

**S3 Fig. Adiponectin levels as functions of age, HbA1c, and sVCAM-1 in the controls and patients with type-1 (T1DM) and type-2 (T2DM) diabetes mellitus.** The lines are best fits.
