# Supplementary material for: Subclinical Inflammation and Endothelial Dysfunction in Young Patients with Diabetes: A Study from United Arab Emirates
Source: PLoS One. 2016 Jul 26;11(7):e0159808. doi: 10.1371/journal.pone.0159808 (PMC4961363; doi:10.1371/journal.pone.0159808)
Supplement: S1 File — (DOCX) [file pone.0159808.s004.docx]

**S4 File. Schematic presentation of inflammatory and endothelial dysfunctions in young patients with diabetes.**
